# Nosocomial COVID-19 Incidence and Secondary Attack Rates among Patients of Tertiary Care Center, Zurich, Switzerland

**DOI:** 10.3201/eid2810.220321

**Published:** 2022-10

**Authors:** Aline Wolfensberger, Verena Kufner, Maryam Zaheri, Marius Zeeb, Isabelle Nortes, Peter W. Schreiber, Miriam Vazquez, Verena Schärer, Thomas Scheier, Stefan Schmutz, Elisabeth Probst, Dirk Saleschus, Michael Huber, Silvana K. Rampini, Walter Zingg

**Affiliations:** University Hospital Zürich and University of Zürich Division of Infectious Diseases and Hospital Epidemiology, Zurich, Switzerland (A. Wolfensberger, M. Zeeb, P.W. Schreiber, M. Vazquez, V. Schärer, T. Scheier, D. Saleschus, W. Zingg);; University of Zürich Institute of Medical Virology, Zurich (V. Kufner, M. Zaheri, M. Zeeb, S. Schmutz, M. Huber);; University Hospital Zürich and University of Zürich Department for Internal Medicine, Zurich (I. Nortes, S.K. Rampini);; University Hospital Zürich Clinic of Immunology, Zurich (E. Probst)

**Keywords:** COVID-19, SARS-COV-2, coronavirus disease, severe acute respiratory syndrome coronavirus 2, zoonoses, nosocomial infections, cross-infection, infection control, infectious disease transmission, risk factors, viruses, Switzerland

## Abstract

Of 1,118 patients with COVID-19 at a university hospital in Switzerland during October 2020–June 2021, we found 83 (7.4%) had probable or definite healthcare-associated COVID-19. After in-hospital exposure, we estimated secondary attack rate at 23.3%. Transmission was associated with longer contact times and with lower cycle threshold values among index patients.

Since the beginning of the COVID-19 pandemic, hospitals have introduced infection prevention and control (IPC) measures to protect inpatients from SARS-CoV-2. Despite these precautions, healthcare-associated COVID-19 has affected a notable proportion of hospitalized patients (*1*–*3*). During the second and third waves of COVID-19 in Switzerland, an increasing number of patients with presymptomatic or asymptomatic COVID-19 exposed hospital roommates to SARS-CoV-2. We estimated the secondary attack rate (SAR) after exposure in a hospital in Zurich and identified risk factors for SARS-CoV-2 transmission.

## The Study

University Hospital Zurich, a 900-bed tertiary care center, implemented intensified standard precaution measures during the COVID-19 pandemic (Appendix). We included in our analysis all patients with COVID-19 admitted during the second and third COVID-19 waves, weeks 40 of 2020 through 25 of 2021; a small percentage were also included in a study investigating transmission to healthcare workers (*4*). We stratified COVID-19 sources as community-associated, healthcare-associated (definite and probable), or indeterminate according to European Centre for Disease Prevention and Control criteria (*5*). We defined index patients as those who, during the 48 hours before onset of signs and symptoms or a positive SARS-CoV-2 test, had contact with an exposed patient. We defined exposed patients as those sharing a room with an index patient for >6 hours in an intermediate care unit (IMC) or intensive care unit (ICU) (*1*), any time on the general ward, or when the index patient underwent an aerosol-generating procedure (*2*). We initiated droplet isolation precaution measures for exposed patients and tested them upon symptom onset or, beginning week 47 of 2020, systematically at 2, 5, and 10 days after exposure. We contacted discharged patients by phone and offered testing in the outpatient clinic. Patients with up-to-date vaccination or known COVID-19 during the previous 6 months were considered unexposed. 

We assessed transmission pathways between patients by in-depth reviews of symptom onset and dynamics of cycle threshold (Ct) values. If sequencing results were available, evidence of transmission was defined as ≤1 single-nucleotide polymorphism (SNP). Exposed patients with >1 SNP difference from the index patient were excluded from the main analysis, but patients without sequencing data were included. For association with SARS-CoV-2 transmission, we assessed index patient age, sex, aerosol generating procedures, and Ct values from first positive PCR test, duration of contact between an index patient and exposed patient, ward type, and pandemic week. 

Routine SARS-CoV-2 PCR testing was conducted in 3 laboratories and whole-genome sequencing performed according to nCoV-2019 sequencing protocol v3 (LoCost) V.3 (https://www.protocols.io/view/ncov-2019-sequencing-protocol-v3-locost-bp2l6n26rgqe/v3) (Appendix). To estimate SAR, we calculated cumulative incidence using the Kaplan-Meier estimator. We assessed risk factors for transmission in univariate and multivariable logistic regression models. We conducted sensitivity analyses on patients with >10 days clinical or laboratory follow-up (Appendix Table 1), on all patients irrespective of phylogenetic results (Appendix Table 2), and on patients with phylogenetically proven transmission (Appendix Table 3). We conducted analyses using Stata statistical software release 16 (StataCorp LLC, https://www.stata.com) and R version 4.0.2 (https://cran.r-project.org/bin/windows/base). The Zurich Cantonal Ethics Commission waived formal ethics evaluation because our analysis was part of an outbreak investigation for quality control and infection prevention (Req 2021-00560). 

**Figure 1:**
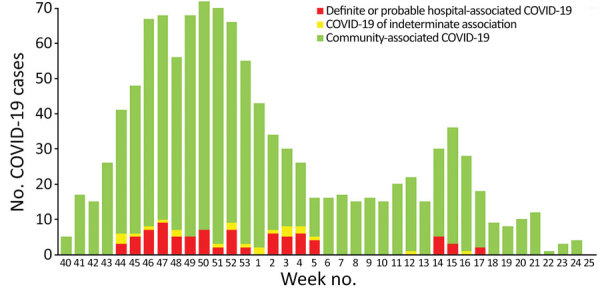
Incidence of admitted patients with positive SARS-CoV-2-PCR per week, including categorization hospital-associated versus community-associated, temporal trend of incidence of SARS-CoV-2 positive patients from week 40 of 2020 through week 25 of 2021. Incident cases were stratified according to European Centre for Disease Prevention and Control definitions of healthcare-associated COVID-19.

**Figure 2:**
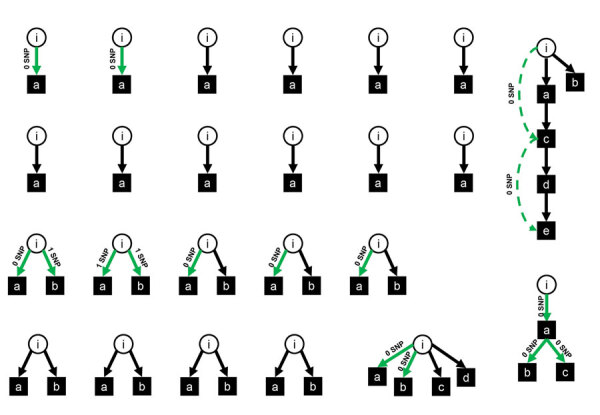
Transmission clusters of patients after exclusion of 5 exposed patients in whom phylogenetic data did not support transmission. Circles are index patients, squares are infected contact patients. Green arrows represent phylogenetically confirmed transmissions, with the labels “0 SNP” and “1 SNP” indicating 0 or 1 SNP difference between index and exposed patient. Green dashed arrows represent phylogenetic proof of second-generation transmission. Black arrows represent assumed transmissions without phylogenetic proof. i, index patient; a–d, exposed

Of 1,118 patients with COVID-19, a total of 1,012 (90.5%) cases were community-associated, 40 (3.6%) probable healthcare-associated, 43 (3.8%) definite healthcare-associated, and 23 (2.1%) indeterminate (Figure 1). In total, we found 127 index patients for 303 exposed patients. Phylogenetic data supported transmission in 14/23 pairs of index–exposed patients with epidemiologic links for whom we had available data (Appendix Figure 1). In addition, we confirmed 4 transmissions indirectly by data from transmission chains (Figure 2). We excluded 5 exposed patients from the analysis because of >1 SNP difference between index and exposed patient. Among exposed patients in the analysis, 42/298 (14.1%) tested positive for SARS-CoV-2 and 179/298 (69.9%) had a follow-up time <10 days. Cumulative incidence for COVID-19 as an estimator of the SAR was 23.3% (95% CI 16%–30%) (Appendix Figure 2). Clusters were small, with only 2 multigeneration transmissions (Figure 2). We found links to identified index patients for 26 (65%) patients with probable and 15 (34.9%) patients with definite healthcare-associated COVID-19. 

We performed univariable and multivariable analyses to explore factors associated with transmission of SARS-CoV-2 from patients to roommates (Table). We found similar results for 2 of the 3 sensitivity analyses (Appendix Tables 2, 3). However, in analysis of patients with a follow-up ≥10 days (Appendix Table 1), exposure on IMC/ICUs and higher number of weeks into the COVID-19 pandemic were associated with a lower risk for transmission, likely because of greater physical distance between immobile patients on the IMC/ICU and increased IPC standards. 

**Table:** Univariable and multivariable analysis of factors associated with SARS-CoV-2 transmission to exposed patients in a hospital in Zurich, Switzerland, October 2020–June 2021*

Exposure	Exposed patients positive for SARS-CoV-2, n = 42	Exposed patients not positive for SARS-CoV-2, n = 256	OR (95%CI)	
			Univariable analysis	Multivariable analysis
Contact time of index and exposed patient in hours, median (IQR)	54 (28–96)	17 (8–29)	1.03 (1.02–1.03)	1.02 (1.01–1.03)
Ct value of index patient in units, median (IQR)	19 (18–26)	28 (19–33)	0.91 (0.87–0.96)	0.93 (0.87–0.98)
AGP in index patient, mean (SD)	0.26 (0.44)	0.25 (0.43)	1.04 (0.50–2.19)	NA
Exposure on IMC/ICU, mean (SD)	0.14 (0.35)	0.31 (0.46)	0.37 (0.15–0.92)	0.70 (0.27–1.87)
Male sex of index patient, mean (SD)	0.55 (0.50)	0.52 (0.50)	1.08 (0.56–2.09)	NA
Age of index patient, y, median (IQR)	71 (58–77)	72 (58–78)	1.00 (0.98–1.02	NA
Exposure before mandatory patient masking at bed place, mean (SD)	0.09 (0.28)	0.11 (0.32)	1.41 (0.50–3.96	NA
Calendar week into second and third waves, median (IQR)	13 (9–17)	13 (8–18)	0.96 (0.91–1.01)	0.95 (0.90–1.01)

*AGP, aerosol-generating procedures; Ct, cycle threshold; IQR, interquartile range; IMC, intermediate care units; ICU, intensive care units; NA, not available; OR, odds ratio.

In a mostly unvaccinated population in which most infections were caused by pre-Alpha variant SARS-CoV-2 (*6*), we found that 7.4% of all COVID-19 patients had probable or definite healthcare-associated COVID-19. This finding is comparable to that from the second wave in Brazil (8.6%) (*7*) but lower than that from the first wave in the United Kingdom (9%–15%) (*2*,*3*). We were able to link only half of the healthcare-associated cases in our hospital to an identified index patient. Despite all the IPC measures in place, high population incidence probably contributed to an increased risk for healthcare-associated transmissions from other patients but also from visitors and HCWs. 

We identified ≈11% of all COVID-19 patients as index patients and estimated a 23% SAR in exposed patients. From the 23 epidemiologically linked pairs with available phylogenetic data, transmission was endorsed in only 18, suggesting that the overall index-to-contact patient transmission rate may have been overestimated. SARs among hospital roommates in a study from a tertiary care center in Iowa, USA, was 21.6% (*8*); from a tertiary care center in New York, New York, USA, 18.9% (*9*); and from a hospital in Boston, Massachusetts, USA, 39% (*10*). These SAR numbers are comparable to those in households (*11*), implying either that distancing and masking are of limited effectiveness for preventing transmission while sharing accommodations (supporting an aerosol transmission pathway between patients) (*13*) or that adherence to distancing and masking were low. Unsurprisingly, as also demonstrated elsewhere (*8*,*10*), the 2 parameters most strongly associated with SARS-CoV-2 were longer contact time between index and exposed patients and low Ct values (i.e., high viral loads) among index patients; Ct values <21 were shown to be associated with transmission (*10*). 

Among limitations in our study, phylogenetic results were available for only half of the patients, laboratory follow-up with inpatients was only 10 days, and discharged patients were often not available for further follow-up. We also limited contact time on IMC/ICUs to >6 hours, which might have excluded relevant contacts, and we might have missed superinfection. Finally, we were unable to model potential drivers for transmission, such as patient nonadherence to IPC-measures, distance between index and exposed patients, or respiratory signs or symptoms of index patients. 

## Conclusions

High viral loads among index patients and prolonged contact time in shared hospital rooms play critical roles in healthcare-associated SARS-CoV2-transmission. Although based on data from a time when pre-Alpha and Alpha variants circulated in a nonvaccinated population, our findings might be relevant in the context of more recently emerged and future variants of concern (*13*,*14*) and waning immunity (*15*). The findings in our study and other studies of substantial SARs in hospitals support early adoption strategies to prevent healthcare-associated transmission during times of high population COVID-19 incidence. Those strategies include identifying contagious patients early (e.g., by performing systematic and repetitive SARS-CoV-2 testing), improving mask-wearing adherence in patients, and frequently replacing air in shared patient rooms. 

Appendix Additional information on study of nosocomial COVID-19 incidence and secondary attack rates among patients of tertiary care center in Zurich, Switzerland

## References

[1] Luong-Nguyen M, Hermand H, Abdalla S, Cabrit N, Hobeika C, Brouquet A, et al. Nosocomial infection with SARS-Cov-2 within departments of digestive surgery [in French]. J Chir Visc Surg. 2020;157:S13–9. 10.1016/j.jviscsurg.2020.04.016 PMC7183971 32381426

[2] Rickman HM, Rampling T, Shaw K, Martinez-Garcia G, Hail L, Coen P, et al. Nosocomial transmission of coronavirus disease 2019: a retrospective study of 66 hospital-acquired cases in a London teaching hospital. Clin Infect Dis. 2021;72:690–3. 10.1093/cid/ciaa816 32562422 PMC7337682

[3] Taylor J, Rangaiah J, Narasimhan S, Clark J, Alexander Z, Manuel R, et al. Nosocomial COVID-19: experience from a large acute NHS Trust in South-West London. J Hosp Infect. 2020;106:621–5. 10.1016/j.jhin.2020.08.018 32841703 PMC7443059

[4] Zeeb M, Weissberg D, Rampini SR, Müller R, Scheier T, Zingg W, et al. Identifying risk contacts for SARS-CoV-2 transmission to healthcare-workers during a COVID-19 ward outbreak. Emerg Infect Dis. 2022. In press. 10.3201/eid2810.220266 PMC9514331 36001791

[5] European Centre for Disease Prevention and Control. Surveillance definitions for COVID-19: source of infection: healthcare (nosocomial) vs community transmission [cited 2022 Aug 16] https://www.ecdc.europa.eu/en/covid-19/surveillance/surveillance-definitions

[6] Federal Office of Public Health Switzerland. COVID-⁠19 Switzerland information on the current situation [cited 2022 Aug 16]. https://www.covid19.admin.ch/en

[7] Tauffer J, Konstantyner TCRO, de Almeida MCS, Ferreira DB, Antonelli TS, Fram DS, et al. Impact of In-Hospital infection with SARS-CoV-2 among Inpatients at a university hospital. Am J Infect Control. 2021;49:1464–8. 10.1016/j.ajic.2021.09.015 34551334 PMC8451472

[8] Trannel AM, Kobayashi T, Dains A, Abosi OJ, Jenn KE, Meacham H, et al. Coronavirus disease 2019 (COVID-19) incidence after exposures in shared patient rooms in a tertiary-care center in Iowa, July 2020-May 2021. Infect Control Hosp Epidemiol. 2021;•••:1–4. 10.1017/ice.2021.313 34250882 PMC8326672

[9] Chow K, Aslam A, McClure T, Singh J, Burns J, McMillen T, et al. Risk of healthcare-associated transmission of SARS-CoV-2 in hospitalized cancer patients. Clin Infect Dis. 2022;74:1579–85. 10.1093/cid/ciab670 34329418 PMC8385815

[10] Karan A, Klompas M, Tucker R, Baker M, Vaidya V, Rhee C. The risk of SARS-CoV-2 transmission from patients with undiagnosed Covid-19 to roommates in a large academic medical center. Clin Infect Dis. 2022;74:1097–100. 10.1093/cid/ciab564 34145449

[11] Fung HF, Martinez L, Alarid-Escudero F, Salomon JA, Studdert DM, Andrews JR, et al.; Stanford-CIDE Coronavirus Simulation Model (SC-COSMO) Modeling Group. The household secondary attack rate of severe acute respiratory syndrome coronavirus 2 (SARS-CoV-2): a rapid review. Clin Infect Dis. 2021;73(Suppl 2):S138–45. 10.1093/cid/ciaa1558 33045075 PMC7665336

[12] Greenhalgh T, Jimenez JL, Prather KA, Tufekci Z, Fisman D, Schooley R. Ten scientific reasons in support of airborne transmission of SARS-CoV-2. [Erratum in Lancet. 2021;397:1808.]. Lancet. 2021;397:1603–5. 10.1016/S0140-6736(21)00869-2 33865497 PMC8049599

[13] Ai J, Zhang H, Zhang Y, Lin K, Zhang Y, Wu J, et al. Omicron variant showed lower neutralizing sensitivity than other SARS-CoV-2 variants to immune sera elicited by vaccines after boost. Emerg Microbes Infect. 2022;11:337–43. 10.1080/22221751.2021.2022440 34935594 PMC8788341

[14] Zhang L, Li Q, Liang Z, Li T, Liu S, Cui Q, et al. The significant immune escape of pseudotyped SARS-CoV-2 variant Omicron. Emerg Microbes Infect. 2022;11:1–5. 10.1080/22221751.2021.2017757 34890524 PMC8725892

[15] Poukka E, Baum U, Palmu AA, Lehtonen TO, Salo H, Nohynek H, et al. Cohort study of Covid-19 vaccine effectiveness among healthcare workers in Finland, December 2020 - October 2021. Vaccine. 2022;40:701–5. 10.1016/j.vaccine.2021.12.032 34953607 PMC8683266

