# Supplementary material for: Nosocomial COVID-19 Incidence and Secondary Attack Rates among Patients of Tertiary Care Center, Zurich, Switzerland
Source: Emerg Infect Dis. 2022 Oct;28(10):2087–90. doi: 10.3201/eid2810.220321 (PMC9514341; doi:10.3201/eid2810.220321)
Supplement: Appendix — Additional information on study of nosocomial COVID-19 incidence and secondary attack rates among patients of tertiary care center in Zurich, Switzerland [file 22-0321-Techapp-s1.pdf]

Article DOI: https://doi.org/10.3201/eid2810.220321

Nosocomial COVID-19 Incidence and Secondary Attack Rates among Patients of Tertiary Care Center, Zurich, Switzerland

Appendix

Intensified Standard Precautions during the COVID-19 Pandemic

For HCW:

- Systematic use of surgical masks.
- Wearing protective eyewear when approaching patients not wearing masks.
- Using FFP2 masks during aerosol-generating procedures (AGP).

For patients: Wearing face masks whenever leaving the bed and, from week 45 in 2020, also when staff or visitors were approaching the bed.

In patient rooms:

- Curtains between beds were requested to be kept closed.
- Windows opened regularly in nonventilated rooms after week 47.
- Aerosol-generating procedures other than nebulization in multibed rooms were minimized but not prohibited.

Laboratory Testing and Phylogenetic Analysis

SARS-CoV-2 polymerase chain reaction (PCR) testing was conducted in three different laboratories using either Roche cobas SARS-CoV-2 IVD test (Roche Diagnostics, Mannheim, Germany), Thermofisher Scientific SARS-CoV-2- IVD test (Thermofisher Scientific, Basel, Switzerland), GeneXpert Xpress SARS-CoV-2 (Cepheid, CA, USA), or two in-house RT-PCR assays as described by Corman et al. and Tastanova et al. (*1*,*2*).

SARS-CoV-2 whole-genome sequencing was performed on all available samples according to the nCoV-2019 sequencing protocol v3 (LoCost) V.3 (https://www.protocols.io/view/ncov-2019-sequencing-protocol-v3-locost-bh42j8ye). SARS-CoV-2 consensus sequences were generated by iteratively aligning reads using SmaltAlign (https://github.com/medvir/SmaltAlign), and sequences were uploaded to GISAID. The sequence lineages were obtained using the PANGOLIN package version v1.2.66 (*3*). Background sequences which represent the most frequent lineages during the investigated time interval in the Zurich region were downloaded from GISAID (*4*) and implemented in the phylogenetic analysis. Multiple sequence alignment was done with MAFFT v7.271 (*5*), followed by phylogenetic analysis using RAxML (*6*).

References

1. Corman VM, Landt O, Kaiser M, Molenkamp R, Meijer A, Chu DK, et al. Detection of 2019 novel coronavirus (2019-nCoV) by real-time RT-PCR. Euro Surveill. 2020;25:2000045. PubMed https://doi.org/10.2807/1560-7917.ES.2020.25.3.2000045

2. Tastanova A, Stoffel CI, Dzung A, Cheng PF, Bellini E, Johansen P, et al. A comparative study of real-time RT-PCR-based SARS-CoV-2 detection methods and its application to human-derived and surface swabbed material. J Mol Diagn. 2021;23:796–804. PubMed https://doi.org/10.1016/j.jmoldx.2021.04.009

3. O'Toole A, Scher E, Underwood A, Jackson B, Hill V, McCrone JT, et al. Assignment of epidemiological lineages in an emerging pandemic using the pangolin tool. Virus Evol. 2021;7:veab064.

4. Khare S, Gurry C, Freitas L, Schultz MB, Bach G, Diallo A, et al. GISAID's role in pandemic response. China CDC Wkly. 2021;3:1049–51. PubMed https://doi.org/10.46234/ccdcw2021.255

5. Katoh K, Standley DM. MAFFT multiple sequence alignment software version 7: improvements in performance and usability. Mol Biol Evol. 2013;30:772–80. PubMed https://doi.org/10.1093/molbev/mst010

6. Kozlov AM, Darriba D, Flouri T, Morel B, Stamatakis A. RAxML-NG: a fast, scalable and user-friendly tool for maximum likelihood phylogenetic inference. Bioinformatics. 2019;35:4453–5. PubMed https://doi.org/10.1093/bioinformatics/btz305

Appendix Table 1. Sensitivity analysis S1 including only patients with follow-up of ≥10 days (all patients except those with proven nontransmission), univariable and multivariable analyses of factors associated with SARS-CoV-2 transmission to exposed patients

Exposure	Exposed patients positive for SARS-CoV-2, n = 42	Exposed patients not positive for SARS-CoV-2, n = 77	OR univariable analysis (95% CI)	p value	OR multivariable analysis (95% CI)	p value
Exposure time in hours, median (IQR)	53 (28–96)	17 (11–25)	1.02 (1.01–1.03)	<.001	1.02 (1.00–1.03)	.007
Ct value of index patient in units, median (IQR)	19 (18–26)	27 (20–33)	0.92 (0.87–0.98)	.006	0.93 (0.87–1.00	.039
AGP in index patient, mean (SD)	0.26 (0.45)	0.26 (0.44)	1.01 (0.42–2.38)	.979	NA	NA
Exposure on IMC/ICU, mean (SD)	0.14 (0.35)	0.52 (0.50)	0.15 (0.06–0.41)	.001	0.25 (0.09–0.72)	.011
Male gender of index patient, mean (SD)	0.55 (0.50)	0.62 (0.48)	0.73 (0.34–1.57)	.421	NA	NA
Age of index patient in years, median (IQR)	71 (58–77)	72 (62–78)	0.99 (0.96–1.01)	.627	NA	NA
Exposure before mandatory patient masking at bedplace, mean (SD)	0.12 (0.33)	0.09 (0.29)	1.35 (0.40–4.56)	.511	NA	NA
Calendar week into 2nd and 3rd wave, median (IQR)	13 (9–17)	17 (12–18)	0.92 (0.87–0.98)	.010	0.92 (0.86–1.00)	.045

*Ct, cycle threshold; OR, odds ratio; IQR, interquartile range; IMC, intermediate care unit; ICU, intensive care unit; NA, not available.

Appendix Table 2. Sensitivity analysis S2 including all patients irrespective of phylogenetic result, univariable and multivariable analyses of factors associated with SARS-CoV-2 transmission to exposed patients

Exposure	Exposed patients positive for SARS-CoV-2, n = 47	Exposed patients not positive for SARS-CoV-2, n = 256	OR univariable analysis (95% CI)	p value	OR multivariable analysis (95% CI)	p value
Exposure time in hours, median (IQR)	57 (28–96)	17 (8–29)	1.03 (1.02–1.04)	<.001	1.02 (1.02–1.03)	<.001
Ct value of index patient in units, median (IQR)	20 (18–26)	28 (20–33)	0.91 (0.87–0.96)	<.001	0.93 (0.88–0.98)	.010
AGP in index patient, mean (SD)	0.26 (0.44)	0.25 (0.43)	1.03 (0.50–2.10)	.938	NA	NA
Exposure on IMC/ICU, mean (SD)	0.17 (0.38)	0.31 (0.46)	0.45 (0.20–1.01)	.032	0.88 (0.36–2.12)	
Male gender of index patient, mean (SD)	0.55 (0.50)	0.52 (0.50)	1.13 (0.60–2.11)	.707	NA	NA
Age of index patient in years, median (IQR)	71 (58–78)	72 (58–77)	1.00 (0.98–1.02)	.810	NA	NA
Exposure before mandatory patient masking at bedplace, mean (SD)	0.09 (0.28)	0.11 (0.31)	1.27 (0.45–3.52)	.652	NA	NA
Calendar week into 2nd and 3rd wave, median (IQR)	12 (8–17)	15 (9–18)	0.95 (0.90–0.99)	.022	0.94 (0.89–1.00)	.050

*Ct, cycle threshold; OR, odds ratio; IQR, interquartile range; IMC, intermediate care unit; ICU, intensive care unit; NA, not available.

Appendix Table 3. Sensitivity analysis S3 including only patients with phylogenetically proven transmission, univariable and multivariable analyses of factors associated with SARS-CoV-2 transmission to exposed patients

Exposure	Exposed patients positive for SARS-CoV-2, n = 18	Exposed patients not positive for SARS-CoV-2, n = 256	OR univariable analysis (95% CI)	p value	OR multivariable analysis (95% CI)	p value
Exposure time in hours, median (IQR)	53 (22-82)	17 (8–29)	1.02 (1.01–1.03)	<.001	1.02 (1.01–1.03)	.004
C_t value of index patient in units, median (IQR)	19 (18–20)	28 (20–33)	0.83 (0.76–0.92)	<.001	0.83 (0.75–0.93)	.001
AGP in index patient, mean (SD)	0.11 (0.32)	0.25 (0.42)	0.38 (0.08–1.7)	.199	NA	NA

Exposure	Exposed patients positive for SARS-CoV-2, n = 18	Exposed patients not positive for SARS-CoV-2, n = 256	OR univariable analysis (95% CI)	p value	OR multivariable analysis (95% CI)	p value
Exposure on IMC/ICU, mean (SD)	0.17 (0.38)	0.31 (0.46)	0.44 (0.12–1.56)	.032	NA	NA
Male gender of index patient, mean (SD)	0.56 (0.51)	0.52 (0.50)	1.08 (0.56–2.09)	.811	NA	NA
Age of index patient in years, median (IQR)	70 (58–80)	72 (58–78)	1.00 (0.98–1.03)	.792	NA	NA
Exposure before mandatory patient masking at bedplace, mean (SD)	0.09 (0.28)	0.00 (0.00)	NA	NA	NA	NA
Calendar week into 2nd and 3rd waves, median (IQR)	13 (11–17)	15 (9–18)	0.97 (0.90–1.04)	.330	NA	NA

*C_t, cycle threshold; OR, odds ratio; IQR, interquartile range; IMC, intermediate care unit; ICU, intensive care unit; NA, not available.

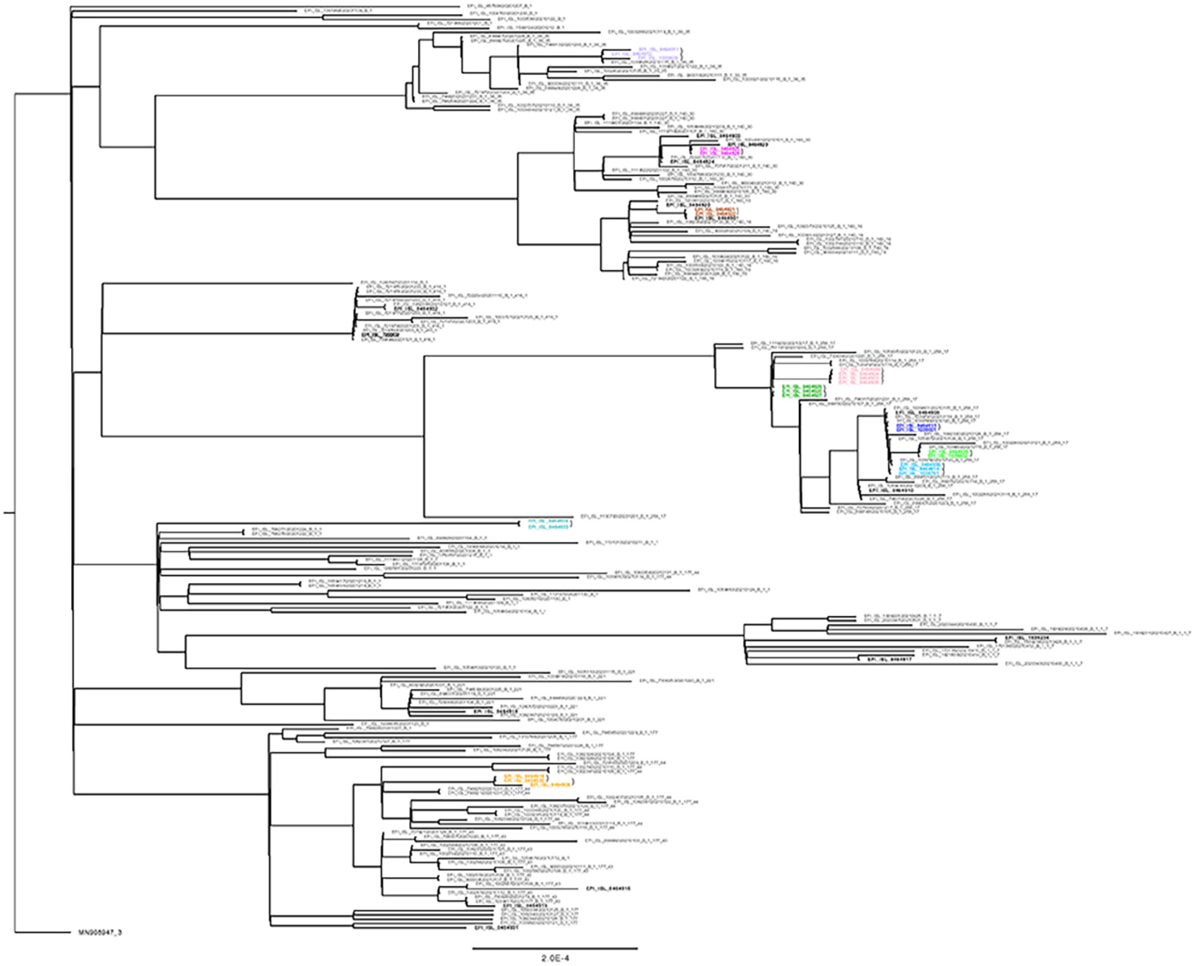

Appendix Figure 1. Phylogenetic analysis of SARS-CoV-2 isolates from 41 patients and 1 healthcare worker, and SARS-CoV-2 background sequences downloaded from GISAID. Patient isolates are indicated using GISAID accession IDs, colors, and parentheses indicate supported transmission clusters.

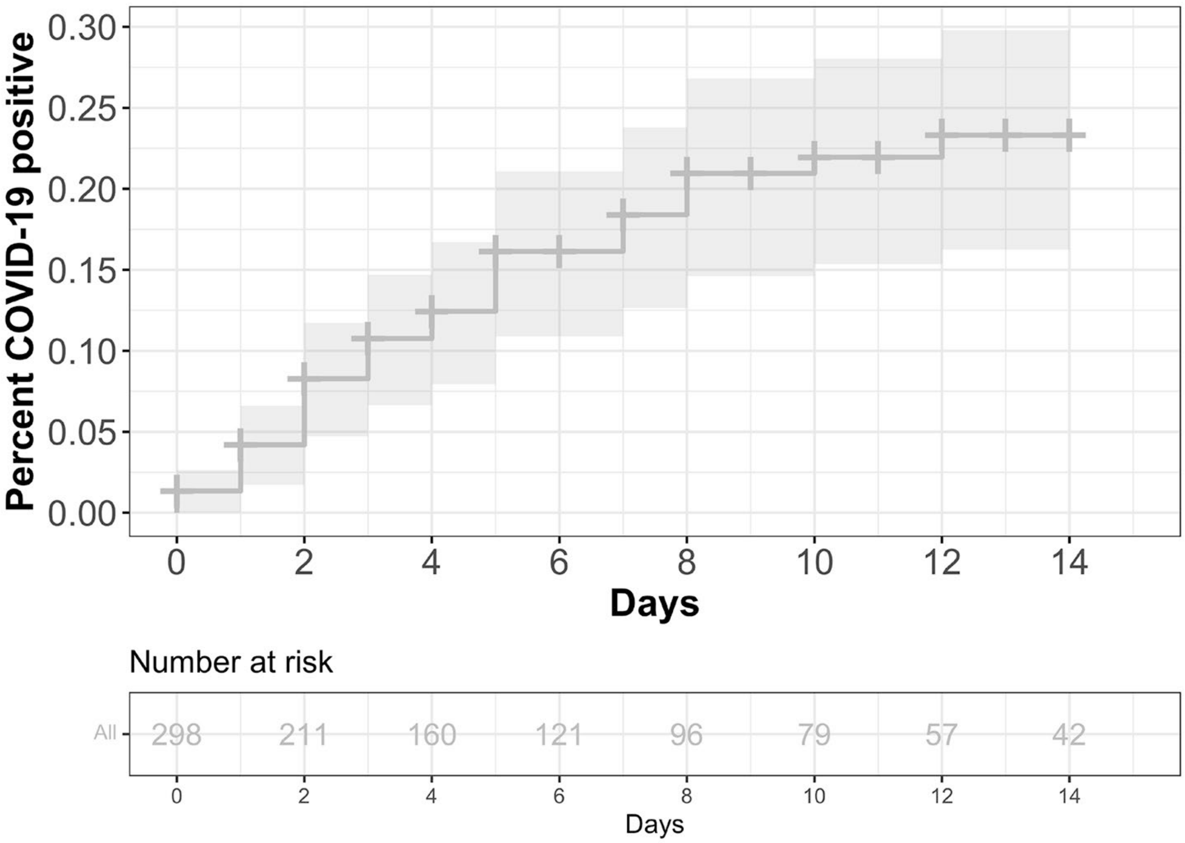

Appendix Figure 2. Cumulative incidence of COVID-19 in exposed patients. X-axis indicates days since last exposure to index case, y-axis indicates percentage of exposed patients who tested positive for SARS-CoV-2, grey-shaded area corresponds to 95% CIs. Bottom graph shows number of patients at risk per day of follow-up after exposure.
